# Cyanotic Raynaud's phenomenon induced by amphotericin B deoxycholate: A case report

**DOI:** 10.1016/j.mmcr.2023.06.003

**Published:** 2023-06-19

**Authors:** Nayla A. Hatem, Taís L. Denicol, Carolina M. Dagostini, Alessandro C. Pasqualotto

**Affiliations:** a Santa Casa de Misericórdia de Porto Alegre, Porto Alegre, Brazil; b Universidade Federal de Ciências da Saúde de Porto Alegre (UFCSPA), Porto Alegre, Brazil

**Keywords:** Cryptococcus, Meningitis, Amphotericin B, Raynaud's phenomenon

## Abstract

A 59-year-old male patient with chronic headache was admitted to the emergency department due to cryptococcal meningitis. His past medical history was marked by liver transplant 18 months prior to admission. Induction therapy with amphotericin B deoxycholate was initiated and the patient developed cyanotic Raynaud's phenomenon related to the infusion. The antifungal treatment was switched to liposomal amphotericin B, with complete resolution of the phenomenon.

## 1 Introduction

Amphotericin-B formulations are widely used for the treatment of systemic fungal infections due to their broad antifungal spectrum [1]. However, their use may be limited due to its adverse effects, including infusion-related reactions and renal toxicity. These side effects are particularly seen with the deoxycholate formulation (AmB-d) [2]. Despite being a well-known drug, one side effect of amphotericin B that has been poorly described in the literature is the development of Raynaud's phenomenon.

Raynaud's phenomenon is a vasospastic disorder that primarily affects the extremities, resulting in distinct colour changes. Its prevalence in the general population is estimated to be between 3 and 5% [3]. However, there is limited information in the literature regarding the amphotericin B deoxycholate induced cyanotic Raynaud's phenomenon. Here we report the fourth case of cyanotic Raynaud's phenomenon induced by amphotericin B deoxycholate in the medical literature.

## 2 Case presentation

A 59-year-old male patient presented to the Neurology Outpatient Clinic with a four-month history of mild headache, associated with photophobia and nausea. His past medical history was marked by liver transplant (performed 18 months prior to this medical appointment) due to hepatitis B virus-associated hepatocellular carcinoma. The patient was under post-transplant immunosuppressive treatment with tacrolimus 1 mg bds and everolimus 0.75 mg bds.

Physical evaluation was unremarkable and a primary investigation with magnetic resonance image showed no pathological changes. Four months later, the patient returned to the clinic reporting persistence of the headache with increase in its intensity for the previous four weeks, associated with neck pain and a few isolated fever episodes. He reported that symptoms did not relieve with over-the-counter analgesics. Besides, the patient reported poor appetite and unintentional weight loss (∼8 kg), during the same period. Neurological examination did not show focal symptoms and there was no signs of meningeal irritation.

A lumbar puncture was performed, and cerebral spinal fluid analysis revealed 69 white blood cells (97% lymphocytes), protein level of 132 mg/dL and glucose level of 18 mg/dL. Opening pressure was 50 mmHg. Cryptococcal antigen was positive in the cerebral spinal fluid, and the patient was referred to the Emergency Department (ED) immediately due to cryptococcal meningitis.

On the day of hospital admission [day 0], the patient had no fever, headache and the neurological exam was intact. As a consequence of the bureaucratic processes involved in acquiring flucytosine (5-FC) and liposomal amphotericin B, these medications were not readily accessible. Consequently, the patient's treatment for cryptococcal meningitis commenced with monotherapy using intravenous AmB-d (0.7 mg/kg daily) for the induction therapy. Despite the potential for concurrent use of fluconazole and tacrolimus with appropriate dose adjustment, as a consensus with our transplant team, combined fluconazole was not initiated.

**Fig. 1:**
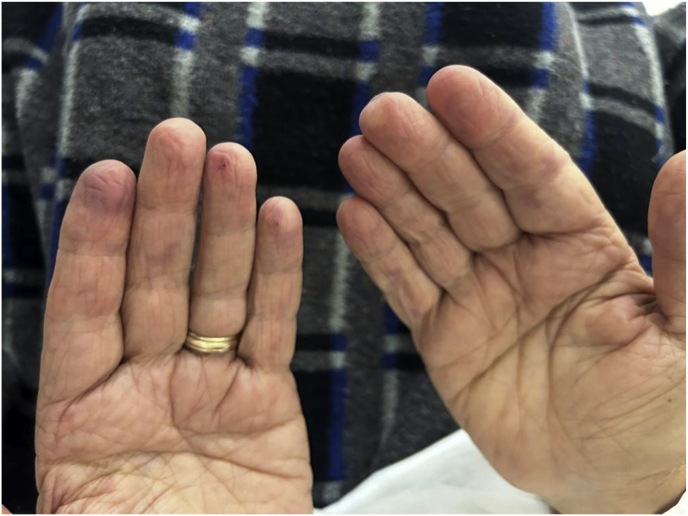
Cyanosis of fingers developed after amphotericin B deoxycholate use.

Three days later [day 3] the patient developed heterochromia on his fingers and feet (mixed areas of pale, hyperaemia, and cyanosis), associated with paraesthesia and pain (Fig. 1). He noticed these symptoms within 30 minutes of the anti-fungal infusion and reported complete relieve 2 h later. He denied previous episodes of Raynaud's phenomenon before the admission. After the successful acquisition and dispensation of the medications by the hospital, AmB-d treatment was discontinued [day 4], and induction therapy was switched to liposomal amphotericin B (3 mg/kg/daily) plus flucytosine (100 mg/kg/daily in 4 divided doses). He completed two weeks of induction therapy, and no more episodes of cyanosis were reported.

## 3 Discussion

Amphotericin B is a key drug in the antifungal armamentarium and is frequently used for the treatment of life-threatening fungal infections in immunocompromised patients [1]. However, its use may be accompanied by toxicities, with a high incidence of infusion-related reactions and nephrotoxicity. These toxicities are most commonly associated with AmB-d [2]. The authors report the fourth case in medical literature of cyanotic Raynaud's phenomenon associated with AmB-d.

Raynaud's phenomenon is defined as a vasospasm of the arteries and arterioles inducing colour changes of the extremities [3]. It is a transient, symmetric, and at times painful acral condition, which the typical tricolour syndrome is characterized by pallor (vasospasm and decreased blood flow), cyanosis (deoxygenation of the static venous blood) and rubor (reperfusion) [4,5]. It is most prevalent in females, and it is characteristically triggered by cold, fear and stress [3,4].

Based on the aetiology, the Raynaud's phenomenon can be classified as primary (idiopathic) or secondary. This second group is associated with an underlying cause or exposure to medicinal products, including chemotherapy regimens or β-adrenergic receptor blockers [4,5]. Although the mechanisms of drug-related Raynaud's phenomenon are unclear, drugs which peripheral vascular effects leads to decreased microvascular perfusion and may induce or aggravate Raynaud's phenomenon [4]. The real incidence of drug-induced Raynaud's phenomenon is probably not recognized because of the limited knowledge of this side effect.

Three previous cases of Raynaud's phenomenon induced by amphotericin B deoxycholate have been published. All the patients developed cyanosis after AmB-d but were reported to tolerate liposomal amphotericin well [6]. Even though the mechanism is not well-understood, it is believed to be related to the medication's effect on vascular tone, as described on renal toxicity (the most common side effects of Amb-d). The acute renal injury secondary to Amb-d is secondary to a poorly understood toxic effects to the afferent arterioles and tubules. This phenomenon leads to direct renal and systemic vasoconstriction, reducing renal blood flow [7]. The systemic vasoconstriction is hypothesized to happen in the small blood vessels of the fingers and toes, leading to Raynaud's phenomenon.

Since the vasoconstrictive effect of amphotericin B is dose-dependent and is probably mediated by the vessels’ endothelium, a lipid-based formulation could prevent these effects [6]. Liposomal amphotericin B has a significantly improved toxicity profile compared with conventional AmB-d [8]. As a lipid-based formulation, the liposomes do not specifically target fungal cells and this causes slower delivery of the drug to tissues, reducing the toxicity [1,9].

## 4 Conclusion

Cyanotic Raynaud's phenomenon is a rare but possible side effect of AmB-d. Although the mechanism is not well-understood, a lipid-based formulation of amphotericin B may prevent this effect.

## Conflict of interest

Dr Pasqualotto has received research grant support from Gilead, 10.13039/100004319Pfizer, and MSD. He has given paid talks on behalf of Gilead, United Medical/Knight, Pfizer, MSD, IMMY, and Astellas. Dr Hatem, Dr Denicol and Dr Dagostini have no conflict of interest to declare.

## Ethical form

The authors have obtained written and signed consent from the patient to publish the case report.
